# Effect of active learning using program visualization in technology-constrained college classrooms

**DOI:** 10.1186/s41039-015-0014-0

**Published:** 2015-07-31

**Authors:** Gargi Banerjee, Sahana Murthy, Sridhar Iyer

**Affiliations:** 1 Interdisciplinary Program in Educational Technology, Indian Institute of Technology Bombay, Mumbai, 400076 India; 2 Department of Computer Science and Engineering, Indian Institute of Technology Bombay, Mumbai, 400076 India

**Keywords:** Program visualization, Active learning, Engagement levels, Prediction, Viewing

## Abstract

Multiple studies report that Computer Science (CS) instructors face problems on how to integrate visualizations in their teaching. This problem gets compounded for instructors in technology-constrained classrooms that are common in developing countries. In these classrooms, students are not able to interact with visualization directly; instead, their interaction is mediated by the instructor who alone may have access to the visualization. In the current study, we contrasted learning outcome from integrating program visualization at two different engagement levels in instructor-mediated classroom setting. The two levels were “Responding” (prediction activity with visualization) and “Viewing” (watching visualization with instructor commentary) as per Naps’ taxonomy. The study was conducted for a programming topic of medium complexity. We found the strategy of prediction with visualization (“Responding”) led to statistically significant higher active behavioral engagement and higher perception of learning among students than the strategy of watching the visualization with instructor commentary (“Viewing”). We also found statistically significant higher cognitive achievement in terms of the rate of problem solving for the “Responding” group, if the students had prior training in active learning. This study can serve as a reference guide to design effective integration of visualizations in instructor-mediated classrooms.

## Background

**Fig. 1:**
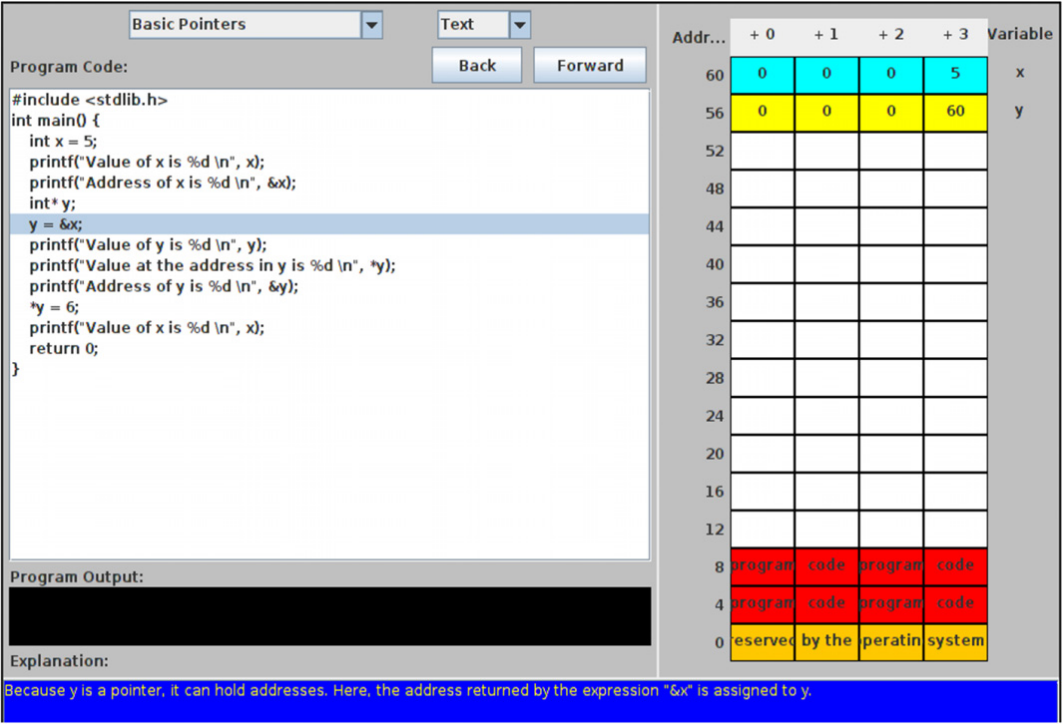
Screenshot of example program visualization (PV) used in current study (Student project and University of Pittsburg 2012)

Computer-based visualizations involve “the use of computer supported, interactive, visual representations of data to amplify cognition” (Tory and Moller 2004) like educational animations and simulations. Well-designed visualizations with affordances that are known to promote learning, like variable manipulations, dynamic multiple representations, and others, have been shown to be effective learning resources (Linn and Eylon 2006). They make the invisible visible and improve prediction and reasoning abilities (Riess and Mischo 2010). In the domain of Computer Science (CS), such visualizations entail the use of graphical entities to depict runtime behaviors of code segments, consequent changes occurring within the computer system or algorithm executions. The visualizations used in CS are classified into two broad categories, based on their level of abstraction—algorithm visualization (AV) and program visualization (PV) (Price et al. 1993). The current study focuses on PV, which is visualization of actual program code at a low level of abstraction, in either static or dynamic form (Price et al. 1993). It includes both animations and simulations (Sorva et al. 2013), depicting the runtime behavior of a program with graphical elements. These graphical elements illustrate the changes occurring within the computer system like changes in the memory map (Fig. 1). These visualizations assist in clearing misconceptions about complex programming topics (Urquiza-Fuentes and Velázquez-Iturbide 2013; Sorva et al. 2013), promote conceptual and procedural understanding (Byrne et al. 1999; Hansen et al. 2000; Laakso et al. 2009), improve verbalization of programming concepts (Ben-Bassat Levy et al. 2003), and understanding the working of the “notional machine” (Sorva et al. 2013) for novice programmers.

However, these benefits from visualizations remain unrealized if instructional strategy used is to simply exhibit the visualization in the classroom (Windschitl and Andre 1998). Such type of passive strategy is prevalent in technology-constrained instructor-mediated classrooms (Kundi and Nawaz 2010). On the contrary, active learning strategies with visualization like prediction activity (Byrne et al. 1999) and peer instruction (Keller et al. 2007) have been shown to lead to positive learning outcome. In fact, Naps et.al. (2002) hypothesized that learning from visualizations will increase across six different student engagement levels with visualization like Viewing (simply watching) to Responding (responding to questions while watching the visualization) to Changing (students manipulating the visualization and seeing its effect). But the results of empirical studies testing Naps’ hypotheses are mixed (Hundhausen and Douglas 2000; Urquiza-Fuentes and Velázquez-Iturbide 2009; Sorva et al. 2013). (In the rest of the paper, we refer to Naps’ engagement levels as “engagement level with visualization” which is different from students’ behavioral engagement). These mixed results point to the impact of moderating variables on the strength of the relationship between the strategy used with visualization and the learning outcome from it.

Multiple studies with CS instructors have revealed that instructors face difficulty on how to integrate visualizations in their teaching (Shaffer et al. 2011). This problem gets compounded for hundreds of instructors in instructor-mediated classrooms, which are the norm in India and a large part of the developing world. The technological resources available in such classrooms are limited to a projector and a laptop with instructors competing for these resources (Kundi and Nawaz 2010). Furthermore, the adoption of technological tools in teaching is not widespread (Mehra and Mital 2007), and the prevailing method of instruction is one-way instructor-centered with preference of text materials over virtual teaching resources (Kundi and Nawaz 2010). Students do not have access to individual laptops, and their interaction with visualization is instructor-mediated. Consequently, the highest possible student engagement level with visualization is constrained to Naps’ “Responding” level. Thus, the challenge for instructors in such classrooms is to design and implement a visualization integration plan that incorporates active learning strategy with visualization to operationalize the “Responding” level of engagement after accounting for the relevant moderating variables.

One proposed solution to tackle the integration problem is sharing of best practices among instructors (Shaffer et al. 2011). Such studies should address a context similar to that of the instructors for whom it is targeted (Prince 2004). Results of the research will then be relevant to instructors since there will be high probability of achieving the same level of learning outcome as reported in the study. The study should also provide exhaustive detail of the visualization integration plan to be meaningful to other instructors. However, most studies on integration of visualizations have either been conducted in a laboratory setting or as a field study measuring the total effect of in-class and lab activities or they invoke the use of additional technological resources like clickers to mediate the interaction (Keller et al. 2007). There are fewer studies that discuss the use of visualizations in an instructor-mediated classroom without access to additional technology. Besides, the majority of the studies with visualization in CS domain have been for AVs and fewer for PVs (also noted by Ben-Ari et al. 2011). These studies can therefore not be taken as integration guides by instructors planning to integrate PVs in instructor-mediated classrooms.

Our study addresses this gap by conducting two field experiments with first-year undergraduate students of CS1 introductory programming course under a condition set typical to instructor-mediated classrooms. It also identifies student’s prior exposure to active learning as a moderating variable and tests for its impact on the learning outcome from visualization by varying this variable across the two experiments. In each experiment, we followed a multi-method research design involving both qualitative and quantitative measures of learning outcome from PV. The qualitative part included classroom observation of students’ behavioral engagement while the quantitative part was a survey to measure student affect and perception of learning. It also included a post-test to measure cognitive achievement. In each experiment, we varied the engagement level with visualization between “Responding” (prediction activity interleaved with watching visualization) and “Viewing” (watching visualization only with parallel instructor commentary) levels for the programming topic of “Pointers.” The students in both groups had no prior knowledge of Pointers. The active learning strategy of prediction with PV was chosen because it was aligned to the instructor’s objectives as well as satisfying instructional requirements like designing a short learning activity targeting close-ended problem solving. To test the impact of the moderating variable-students’ “prior exposure” to active learning on learning outcome, students in experiment II had prior training in active learning while students in experiment I did not. By “prior” exposure, we mean students who have been extensively trained in active learning such that they are accustomed to the mechanics of the process and are tuned to their role in it. To ensure that the student samples had the intended level of “prior exposure,” we conducted checks during sample selection through formal instructor interviews and observations of student behaviors in class as well as informal student interviews. The details of the sample selection process are given in Sections 5.1 and 6.1.

We found the active learning strategy of “Prediction with PV” resulted in significant increase in learning outcome in terms of student behavioral engagement and also on their perception of learning effectiveness and satisfaction of the strategy. Prior exposure to active learning does not appear to be a moderating variable for these metrics. However, we found prior exposure does have a moderating influence on the rate of problem solving that was taken as a measure of cognitive achievement. These results are valid for our condition set of instructor-mediated classroom and medium complexity programming topic in lecture setting. Detailed description and findings of experiments I and II are given in Sections 4, 5, and 6.

The results from our study provide evidence of feasibility and advantage of implementing active learning strategies with visualization in instructor-mediated situations. The study also provides a detailed visualization integration plan for “Prediction with PV” strategy along with the condition set under which these results hold. Previous studies with PVs have identified moderating variables like topic complexity (Urquiza-Fuentes and Velázquez-Iturbide 2013) and student characteristics like achievement level (Ben-Bassat Levy et al. 2003), besides engagement level with visualization, as having an impact on learning outcome from PV. Our study adds to this research on teaching with visualizations by identifying the student characteristic of “prior exposure” to active learning with PV as another moderating variable to consider. It also provides researchers with evidence that students at the “Responding” level exhibited significantly better cognitive achievement over the students at the “Viewing” level if the topic is of medium complexity and the setting is a lecture class in an instructor-mediated environment. The field experiment aspect of the current study provides the necessary ecological validity to the findings.

## Theoretical background and related work

In this section, we focus on the existing work done to test learning outcome from program and algorithm visualizations in response to differing engagement levels with visualization, operationalized by different instructional strategies with visualization. We describe key theories on the effect of engagement level with visualization on learning outcome followed by literature survey of positive and negative empirical studies on CS topics. We also report studies on the students’ behavioral engagement while viewing visualizations and conclude with studies reporting moderating variables that affect learning from visualization.

### Theoretical background

From their meta-analysis of learning effectiveness studies for visualization in CS, Hundhausen et.al. (2002) postulated that how students interact with visualization has a significant impact on their learning from visualization. Based on this, Naps et.al (2002) proposed a taxonomy of six engagement levels for algorithm visualizations-No Viewing, Viewing, Responding, Changing, Constructing and Presenting-hypothesizing that learning will increase as the engagement level with visualization proceeds from “No Viewing” to “Presenting.” Thus, the “Responding” level was hypothesized to lead to better learning outcome with visualization than the “Viewing” level. In the “No Viewing” level, no visualization is involved. In the “Viewing” level, students simply watch the visualization. In the “Responding” level, students not only watch but also interact with the visualization by responding to the visual cues presented like answering exercise or prediction questions. In the “Changing” level, students interact with visualization by changing variable parameters. In the “Constructing” level, students create their own visualization whereas in “Presenting” level, they present their own visualizations to their peers. Myller et al. (2009) added four additional levels and termed these as the “Extended Engagement Taxonomy” (EET). Thus, the ten levels became “No Viewing,” “Viewing,” “Controlled Viewing,” “Entering Input,” “Changing,” “Modifying,” “Constructing,” “Presenting,” and “Reviewing” where “Controlled viewing” means students have control over navigation through the visualization. EET hypothesized that along with learning, collaboration among students will also increase with increasing levels of engagement. Sorva et al. (2013) proposed the 2DET engagement taxonomy consisting of two dimensions of direct engagement with visualization and content ownership (cognitive engagement). The 2DET hypothesizes that learning from program and algorithm visualizations increases along both the axes of direct engagement level and content ownership. Among all the engagement taxonomies with program and algorithm visualizations, Naps’ engagement levels with visualization have historically been one of the most explored conditions while measuring learning from visualizations. Naps’ hypotheses have been tested by multiple studies, but the results are mixed.

### Empirical studies testing Naps’ hypothesis

Numerous studies have been done to test these hypotheses by contrasting learning at multiple levels of student engagement with program and algorithm visualizations. Among the studies confirming Naps’ hypothesis, Grissom et al. (2003) found learning gain increased with increasing student engagement for simple sorting algorithms (insertion and bubble sort) across “No viewing,” “Viewing,” and “Responding” through online quiz. Similar result was reported by Hansen et al. (2000) where instructional strategy used for the “Responding” level was interactive prediction and question-answering. Byrne et al. (1999) did a controlled experiment with CS majors who had algorithm analysis skills but no prior knowledge of the topic, binomial heap. These students did better in procedural understanding in post-test when at the “Responding” level (viewing with oral prediction) or “Viewing” level compared to the “No viewing-No prediction” group. However, the effect of visualization and prediction could not be isolated. Ben-Bassat Levy et al. (2003) did a field study at school level on programming topics like if while statements with the post-test containing questions on predicting output of a program code using Jeliot. They found significant learning gain for all students irrespective of their achievement level with average students gaining the most. Laakso et al. (2009) found learning gain for conceptual understanding at both “Viewing” and “Changing” levels with statistically significant gain at the “Changing” level on the topic Binary heap. However, this result was obtained only after correction for behavioral engagement of student pairs since all students did not perform at the expected level of engagement with visualization. Myller et al. (2009) tested their EET hypothesis and found strong correlation between behavioral engagement among students in terms of collaborative activity of pair programming and the engagement levels with visualization in EET.

In contrast to the above studies, there are studies that did not find a difference in learning outcome at different engagement levels with visualization. Stasko et al. (1993) did not get any significant difference in procedural understanding between the “No viewing” group and group that could run the visualization on their input data sets (Changing level) on the topic of Pairing heap. A possible reason cited was the visualization design was not suited to novice learners. Jarc et al. (2000) found no difference in learning outcome (conceptual and procedural understanding) between “Responding” and “Viewing” where the “Responding” level was operationalized through automated prediction questions for a set of 11 algorithms. A probable reason given was that students in the “Responding” group adopted trial and error method to proceed with the prediction activity instead of focusing on learning. Hundhausen and Douglas (2000) compared learning at “Constructing” and “Viewing” levels for procedural understanding but did not get any significant difference for the topic of Quick sort.

From the analysis of the above studies, the instructional strategies that have been reported to be successful with program and algorithm visualizations are prediction worksheets with visualization (Ben-Bassat Levy et al. 2003), exercise sheets (Laakso et al. 2009), integrated prediction activity (Hansen et al. 2000), and online quiz (Hansen et al. 2000).

### Factors influencing learning from visualization

Closer analysis of results of empirical studies, similar to those reported above, revealed other factors like topic complexity and learner characteristics, in addition to engagement level with visualization, influences the learning outcome from visualizations.

#### Topic complexity

Jarc et al. (2000) found the “Responding” group performed better on difficult topics (graph search, Heap sort), though not significant. Ben-Bassat Levy et al. (2003) found no effect of visualization on simple topics. Urquiza-Fuentes and Velázquez-Iturbide (2013) found no difference in learning outcome between the three groups at “No viewing,” “Viewing,” and “Constructing” levels when the topic is simple like in-fix operators. For topics of medium complexity like user-defined data types, visualization does show an effect when contrasted with “No Viewing,” though no significant difference occurred between “Viewing” and “Constructing” levels. However, significant difference was obtained when the topic was of high complexity like recursive data types but in favor of the “Viewing” level rather than the “Constructing” level on analysis and synthesis level questions.

#### Learner characteristic

Effect of different learner characteristics on learning outcome from program and algorithm visualizations has been studied. Byrne et al. (1999) varied algorithm analysis skill of learners but did not find any significant effect of this skill on the learning outcome from visualizations. Another often studied learner characteristic is the achievement level. Jarc et al. (2000) found interactive prediction with visualization in a lab setting helped the better students but not the poorer ones. A possible reason given was the poorer students treated the prediction activity as a video game, focusing on being entertained rather than on learning. Ben-Bassat Levy et al. (2003) found mediocre students of tenth grade class gained significantly more on using PVs in lecture and lab classes than high- and low-level students, though they also showed some gain but not significant. They also reported learning gain only from the fifth assignment onwards citing time required by students to get accustomed to working with the PV tool. Isohanni and Knobelsdorf (2011) did a qualitative study of student interaction with the PV tool, VIP. They found students were able to adopt productive ways of using VIP for learning programming concepts only when they were sufficiently familiar with the tool. Besides, students take a long time to adapt to active learning strategies (Niemi 2002). They require training on how to execute active learning like collaborating with an unfamiliar classmate (Seidel and Tanner 2013) or reflecting on their solutions (Niemi 2002).

## Research questions and hypotheses

The broad research question for the study was: How does the learning outcome differ between “Responding” and “Viewing” levels with PV when the setting is an instructor-mediated classroom? To answer this question, we did two control-group field experiments following a multi-method research design. The independent variable was engagement level with visualization varying between the levels of “Responding,” operationalized through prediction activity with PV (experimental group) and “Viewing,” operationalized through watching the visualization with parallel instructor commentary (control group). The dependent variable was learning from PV measured along the metrics of behavioral engagement, affect and perception of learning, and cognitive achievement (rate of problem solving and average post-test scores). The experiments were conducted on the topic of “Pointers” where the learning content was of medium topic complexity. The larger goal of conducting two field experiments was to identify and establish the moderating effect of “prior” training in active learning on the learning outcome from active learning with PV. Thus, in experiment II, we answered the same set of research questions by measuring learning outcome along the three metrics with a student sample who had prior training in active learning.

The three research questions explored in the study were as follows:

- RQ1: Does prediction activity with PV (Responding) lead to higher levels of behavioral engagement than viewing the visualization (Viewing) for a programming topic of medium complexity?
- RQ2: What are the student perceptions about learning effectiveness and satisfaction for the respective strategies used with PV in the classroom?
- RQ3: Does prediction activity with PV (Responding) lead to higher levels of cognitive achievement than viewing the visualization (Viewing) for a programming topic of medium complexity?

The alternative hypothesis, corresponding to RQ1, can be stated as: The “Prediction” group will show higher behavioral engagement in the class compared to the “Viewing” group (H_1___1_). The alternate hypothesis corresponding to RQ2 can be stated as: Student perception of learning effectiveness and satisfaction will be higher for the active learning strategy of prediction with PV compared to simply watching the PV (H_1___2_). Two alternative hypotheses flowing from RQ3 can be stated as follows: (i) Prediction activity interleaved with visualization will lead to higher rate of problem solving than simply viewing the visualization with parallel instructor commentary in a programming class (H_1___3_) and (ii) the “Prediction” group will show higher average post-test scores than the “Viewing” group (H_1___4_).

## Research and method

This section gives an overview of the multi-method research methodology followed across both the experiments. The sample characteristic across the two experiments differed only on the characteristic of prior exposure to active learning. The learning outcome between “Responding” and “Viewing” levels in both experiments was analyzed along the three different metrics of behavioral engagement, student affect and perception, and cognitive achievement. The results from both experiments were compared to analyze the impact of the moderating variable, students’ “prior exposure” to active learning on learning outcome with PV.

### Sample

To test the above hypotheses, samples of first-year undergraduate engineering students were drawn from students enrolled in introductory courses in computer programming at engineering institutes in Mumbai, India. These students had self-declared no prior knowledge of “Pointers.” The sample characteristics in both the experiments differed only on students’ “prior exposure” to active learning. In both experiments, students were divided into two sections by the respective institutes for scheduling reasons. Being field experiments, we had to work with these predetermined groups in both experiments. So, there was no chance of randomizing or matching the two groups in either experiment. However, we did prior testing on a criterion that was crucial to our study, i.e., programming skills. The groups were tested for equivalence on the basis of a prior quiz. Assignment of the treatment (“Prediction” vs. “Viewing”) to the groups in each experiment was done on a random basis. Further details containing justification for varying the student characteristic of “prior exposure” to active learning from experiment I to experiment II and how we implemented the sample selection checks on this parameter are given in subsections 5.4, 5.1, and 6.1, respectively.

### Learning materials

**Fig. 2:**
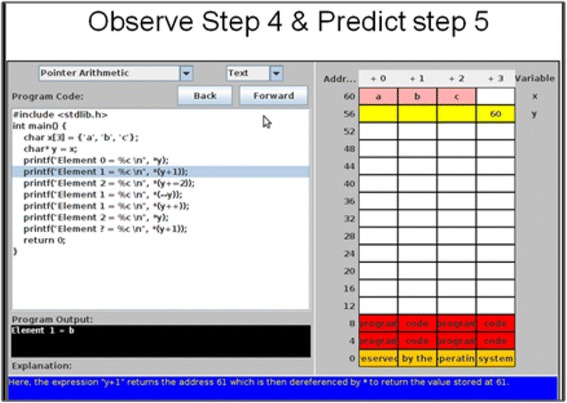
Example prediction activity with PV

The topic “Pointers” deals with variables that store computer memory addresses. This topic was deemed suitable for learning with visualization since it involved making the invisible memory address manipulations visible to the students. The topic was also chosen for its medium topic complexity level. In the absence of a clear definition for topic complexity, we related topic complexity to amount of prior knowledge required to comprehend the topic. The judgment of medium complexity of the learning materials used for “Pointers” was left to the instructor’s content and pedagogical knowledge. We chose medium complexity programming topic since in a prior study with PVs, Urquiza-Fuentes and Velázquez-Iturbide (2013) had found active learning strategy with PV had positive effect on learning of programming topics of medium complexity in contrast to topics of low and high complexity. The PV chosen covered basic pointers and pointer arithmetic with user-controlled navigation. This was PV animation with predefined content (content ownership level in 2DET taxonomy = “Given Content”). The reason for choosing this PV was it satisfied the requirements of visualizations at the “Responding” level as specified by previous research studies (Urquiza-Fuentes and Velázquez-Iturbide 2009) like the presence of explicit feedback and additional narrative or text explanations of what is happening. This visualization displayed the change in memory map in response to execution of each line of code as also its output with explicit explanation of how the output was obtained (Fig. 2).

### Instruments

We did qualitative study for behavioral engagement using a standard observation protocol and used quantitative instruments for measuring affect and perception along with cognitive achievement.

#### Behavioral engagement

Fredericks et al. (2004) categorized engagement studies into three categories—behavioral, emotional, and cognitive. In our study, we measured behavioral engagement of students in terms of student participation in classroom activity with PV. The results of the classroom observations were used to answer RQ1 and test the hypothesis H_1___1_. The observations were based on the standard classroom observation protocol of Behavioral Observation of Students in Schools (BOSS) (Shapiro 2003) (Table 1). It presents a set of codes to analyze student behavior in the classroom into three categories—active engagement, passive engagement, and non-engagement. The in-class observations were coded based on BOSS terminology to report active engagement. For example, behaviors like reading aloud, raising hand, or talking about learning material were coded as active engagement whereas behaviors like listening to lecture/peer answer or reading silently were coded as passive engagement. Some of the non-engagement codes are talking at inappropriate times, manipulating non-related objects and looking around the room. In the current study, we focused on active behavioral engagement. To measure behavioral engagement with the visualization in the classroom, in-lecture observations of students’ behavior were done by multiple researchers in each experiment and inter-coder reliability for the researchers were established through separate pilot studies.

**Table 1:** BOSS Observation protocol (Shapiro 2003). Observation codes

Engagement	
Active engagement	Passive engagement
● Writing	● Listening to lecture
● Reading aloud	● Reading silently
● Raising hand	● Looking at assigned material
● Talking about learning material	● Listening to peer answer related to work
● Talking to peer about learning material	● Looking at locus of attention
Non-engagement (motor, verbal, and passive off-task)	
O—out of seat	S—making audible sounds
MO—manipulating objects non-related	Tl—talking at inappropriate times
TO—touching another students/adult	IR—inappropriate remark
TA—turning his body away/head down; fidgeting in seat	LA—looking around room; staring away

Based on BOSS terminology

#### Affect and perception of learning

The students of both groups answered two questions on a five-point Likert scale questionnaire that captured their perception of the learning effectiveness and also satisfaction of the instructional strategy used with PV in their respective classroom. The student responses on this Likert scale survey were used to answer RQ2 on students’ perceptions on learning through different instructional strategies with PV and test hypothesis H_1___2_.

#### Cognitive achievement

Cognitive achievement was measured in terms of the rate of problem solving and average post-test scores to answer RQ3. The post-test paper contained three post-test questions whose subparts tested conceptual and procedural understanding of basic pointers and pointer arithmetic. The questions were generated by the instructor who was also an educational technology (ET) expert and validated by another ET expert. A sample post-test question is given below.

##### Example post-test question:

Predict the output of the following program:

- int main () {
- int A[4], *p;
- for (int i = 0; i < 4; i++) A[i] = i;
- p = &A[0];
- printf (“ %d %d %d /n” , *p , *(p + =2) ,*(p + 1) + *(p-1));
- return 0;
- }

The total post-test mark was ten marks. Partial marking was done for questions containing subparts. To compute the rate of problem solving, the average time taken to solve the post-test paper for the entire group was recorded.

### Experimental procedure

We did a multi-method research study of student learning with PV. The independent variable was engagement level with visualization. It was varied between the “Responding” level, operationalized through prediction activity with PV (experimental group) and “Viewing” level, operationalized through watching the visualization with parallel instructor commentary (control group). The dependent variable was learning from PV measured along the metrics of behavioral engagement, affect and perception of learning, rate of problem solving, and average post-test scores. The student behavioral engagement was measured through in-lecture observation during the prediction activity with PV for the “Experimental/Prediction” group. For the “Control/Viewing” group, observation was done when the same code segment was shown through the PV.

**Fig. 3:**
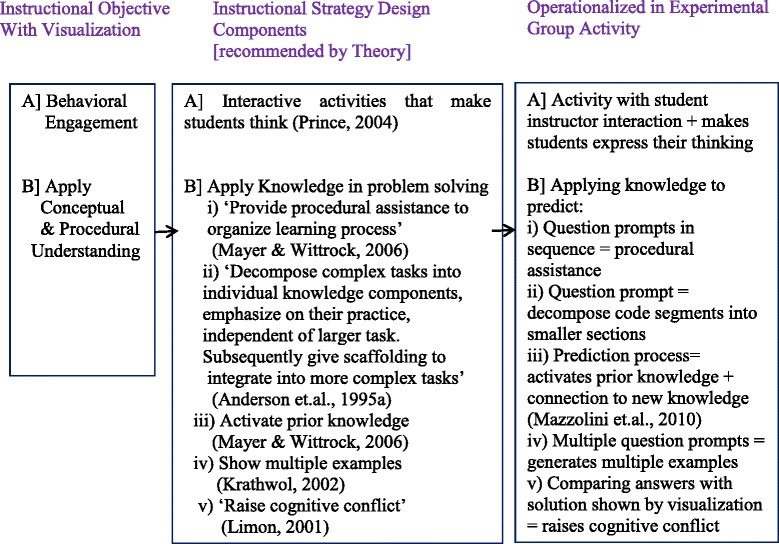
Designing learning activity with instructional strategy design components mapped to objective

**Fig. 4:**
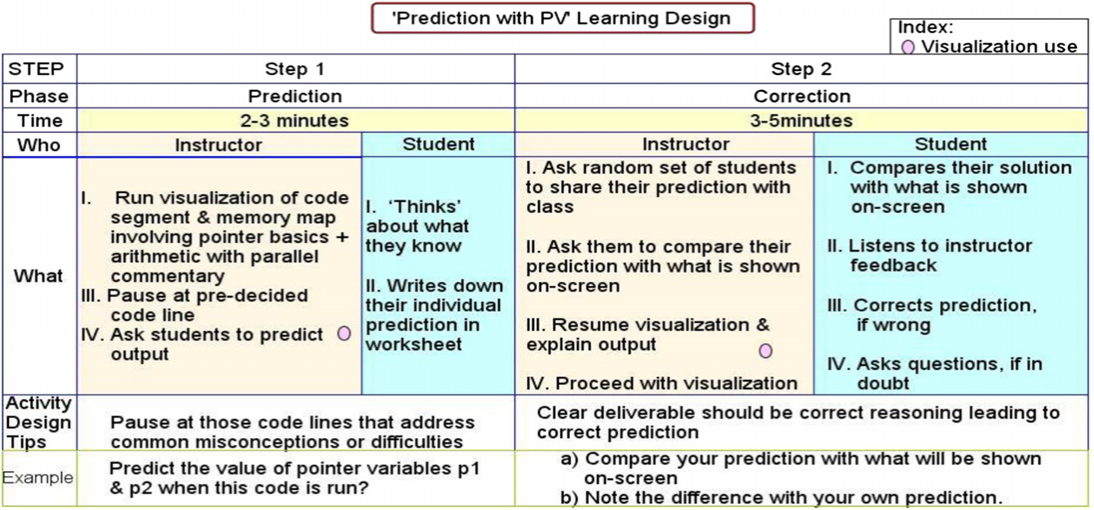
Stepwise implementation plan of “Prediction with PV”

The quantitative part included a two-group post-test only study along with an affect and perception student survey. In the control group experiment, the instructional activity with visualization for the “Prediction” group was designed to include theory-recommended design components that map to our instructional objectives (Fig. 3). The “Prediction” group was given a short theoretical introduction to pointers and pointer arithmetic subtopics during the lecture. In each case, the theoretical explanation was followed by the PV. The PV was run in step-run mode, and students were asked to predict and write down the result of the next step before comparing their answers with what is shown in the visualization. They got immediate, explicit feedback from the PV supplemented with parallel instructor explanation for each step (Fig. 4). Such short prediction activity was done four times within the single code shown in the visualization. We chose the strategy of “Prediction with PV” because it included theory-recommended design components to achieve alignment with our instructional objectives like behavioral engagement of students in the class and application of conceptual and procedural understanding in problem solving (Fig. 3). This strategy also satisfied our requirements of designing a short activity of 10–15 min targeting close-ended problem solving. The prediction activity was followed by the post-test and affect and perception survey.

**Fig. 5:**
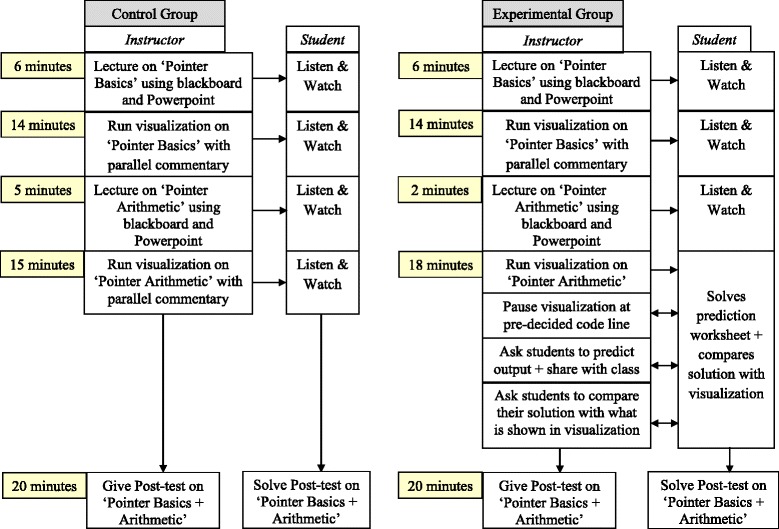
Flowchart of intervention process for the “Prediction” and “Viewing” groups

The “Viewing” group in each experiment was given a longer verbal introduction for the same two subtopics to avoid the confounding variable of two groups having different learning times. The visualization was demonstrated in step-run mode with parallel instructor commentary without explicitly asking the students to make predictions. Both groups were taught by the same instructor with the same lecture content and same PV on the same day with the “Viewing” group going first. The treatment duration was 1 h for both. After the treatment, each group took the same post-test to be solved within a time limit of 20 min followed by the affect and perception survey (Fig. 5).

### Data analysis

The in-class observations of active behavioral engagement for each group, based on BOSS protocol, were tested for significant difference using Pearson’s chi-square test. This test was deemed suitable since both independent (engagement levels with visualization) and dependent variables (behavioral engagement) were categorical with two levels, and the frequency distributions were found to be non-normal from Shapiro-Wilk test. The cell count of the 2 × 2 contingency table was more than 5.

The affect and perception survey responses were analyzed by the non-parametric test of Mann-Whitney *U* to check for significant difference between group responses. This test was chosen since the dependent variable (survey responses) is ordinal and independent variable (treatment) is categorical with two levels. Also, both distributions were found to be non-normal from the Shapiro-Wilk test.

The rate of problem solving is defined as the number of correct responses given to problem solving questions in unit time. To calculate the rate of problem solving for each student (*R*), we divided the post-test score (*C*), representing the number of correct responses of each student in a group, by the average time taken by the group to solve the post-test (*t*), i.e., *R* = (*C*/*t*). The average time of the group was taken since it was not possible to get solving time taken by individual students. Since the distribution of *R* was found to be non-normal by the Shapiro-Wilk test, the non-parametric Mann-Whitney *U* test was done to compare the medians of the two groups. Similarly, the differences between median post-test scores were tested for statistical significance through Mann-Whitney *U* test. This non-parametric statistical test was deemed suitable since the achievement scores showed non-normal distribution as per Shapiro-Wilk test.

## Experiment I

In this section, we describe the sample characteristics and outline the process followed for in-class observation of student engagement distinct to experiment I along with the experimental results.

### Sample characteristics

A sample of 78 first-year undergraduate students was taken. Interviews with a random sample of five of their instructors revealed that these students have never been exposed to active learning strategies in their classroom. They were instead accustomed to the traditional instructor-centered lecture style of teaching with occasional class discussion on instructor-posed questions. This claim was reconfirmed through informal interviews with the students themselves.

Students were divided into two sections by the institute for scheduling reasons. The first section was made the “Prediction” group (*N* = 39; male = 32; female = 7), and the second section was the “Viewing” group (*N* = 39; male = 27; female = 12). The male:female ratio for the “Prediction” (4.6:1) and “Viewing” groups (2.3:1) was comparable. The groups were found to be equivalent on programming skills on the basis of a prior quiz using independent samples *t* test (*M*_experimental_ = 21.21 (SD = 9.94); *M*_control_ = 18.82 (SD = 8.60); *p* > 0.05).

### Procedure for in-class observation

The in-lecture observation of student behavioral engagement was carried out by two observers. Inter-coder reliability for the researchers was established through a separate pilot study with Cohen’s Kappa = 0.64. Each observer observed a random set of 15 students, twice during the prediction activity, for the “Prediction” group and the corresponding code segment for the “Viewing” group using the BOSS codes for active behavioral engagement. Individual students were observed for a fixed time interval of 5 s at a stretch. The total number of observations recorded for each group was 60. The fraction of the class thus observed in each group was 30/39 (76.92 %). The lecture was followed by the post-test and student affect and perception survey.

### Results

#### Behavioral engagement

The observation codes in each group were categorized into engagement (active and passive) and non-engagement as per BOSS protocol. To answer RQ1, we report the percentage of observations out of the total that were in active engagement category (Table 2).

**Table 2:** In-lecture active behavioral engagement with PV

	Prediction group	Viewing group
Total observations	60	60
Active engagement observation frequency (percentage)	24 (40 %)	7 (11.67 %)
Chi-square results	*χ* ^2^ (1) = 17.79, *p* = 0.00	

Forty percent of the “Prediction” group was found to be actively engaged compared to 11.67 % of the “Viewing” group. The chi-square (*χ* ^2^) test on the active engagement observation for each group revealed a significant difference (*p* = 0.00) in favor of the “Prediction” group (Table 2). The prediction activity with PV led to significantly more active behavioral engagement in classroom than viewing alone. Thus, the alternate hypothesis, H _1___1_ stating the “Prediction” group will show increased active behavioral engagement, was accepted.

#### Affect and perception of learning

To answer RQ2, the survey questions asked were as follows: (Q.1) “The instructional strategy used (watching visualization/solving prediction worksheet with visualization) helped me learn.” (Q.2) “I would recommend using the strategy (watching visualization/solving prediction worksheet with visualization) for rest of the course?” In Table 3, we report the total number of responses obtained for each question in the survey. A comparative analysis of the student perception of the strategy used in their respective classrooms was done through the non-parametric Mann-Whitney *U* test results. The result analysis revealed a statistically significant difference in favor of the “Prediction” group for learning effectiveness (*p* = 0.016) and satisfaction (*p* = 0.003) of the “Prediction with PV” strategy. Thus, the hypothesis, H _1___2_ stating there will be higher perception of learning effectiveness and satisfaction for the active learning strategy of “Prediction with PV” compared to simply watching the visualization, is accepted.

**Table 3:** Mann-Whitney *U* test results for affect and perception survey

Question	Group	Mean rank	*U*	*p* values
Q1. Instructional strategy used with visualization helped me learn	Prediction(*N* = 37)	43.11	482	0.016
	Viewing (*N* = 38)	32.18		
Q2. I would recommend using the strategy for rest of the course	Prediction (*N* = 37)	45.24	435	0.003
	Viewing (*N* = 38)	30.95		

#### Cognitive achievement

The “Prediction” and “Viewing” groups were both given 20 min to solve the post-test paper. The analysis of the Mann-Whitney *U* test for both the rate of problem solving and average post-test score did not yield any statistically significant difference between the “Prediction” and “Viewing” groups (Table 4). Thus, the hypotheses, H _1___3_ and H _1___4_ indicating better performance on cognitive achievement for the “Prediction” group vis-à-vis “Viewing” group, could not be accepted.

**Table 4:** Mann-Whitney *U* test for the rate of problem solving and post-test scores

Dimension	Group	Standard deviation	Mean	*U*	*p* values
Rate of problem solving	Prediction (*N* = 39)	0.99	0.23	573	0.12
	Viewing (*N* = 39)	0.12	0.26		
Average post-test score	Prediction (*N* = 39)	1.99	4.54	592	0.09
	Viewing (*N* = 39)	2.42	5.28		

### Discussion of experiment I

From experiment I, we were able to conclude that the instructional strategy of “Prediction” with PV in an instructor-mediated classroom increases students’ active behavioral engagement in a first-year undergraduate introductory programming class compared to simply “Viewing” the PV, when the topic is of medium complexity. Students also perceive the active instructional strategy of “Prediction” with PV to be significantly more likeable and more learning effective than the passive strategy of watching the PV with parallel instructor commentary. These positive results led us to infer that the null result obtained for cognitive achievement may be a function of the students’ first-time exposure to active learning.

In their first exposure, the “Prediction” group performed at par with the “Viewing” group. This finding is backed up by literature evidence that shows sustained exposure to a PV tool led students to devise productive ways to use the tool for learning (Isohanni and Knobelsdorf 2011). Ben-Bassat Levy et al. (2003) also shows learning gains occurring from only the fifth programming assignment onwards, involving both lab and lectures, for the Viewing group (Viewing vs. No viewing). Both these studies involved building up student familiarity with the PV tool during one-to-one interaction. This, along with our results, led us to suspect that there are other factors at play like “prior exposure” to active learning, besides engagement level that influenced learning from PV in the classroom. Therefore, we hypothesized that cognitive achievement (rate of problem solving and average post-test scores) will increase for the “Prediction” group vis-à-vis the “Viewing” group in an introductory programming course if students are given prior training in active learning with PV (H_1___3a_).

To test this hypothesis, we could have trained the same group of students in active learning and carried out further experiments on their learning. But this would have entailed experimenting on different topics of varying complexity. In the absence of clear definitions for topic complexity, this would have inadvertently introduced one more independent variable in the study—topic complexity, which is known to affect learning from program and algorithm visualizations (Urquiza-Fuentes and Velázquez-Iturbide 2013). Hence, we carried out experiment II in which we test the effect of “prior exposure” to active learning on the learning outcome at two different levels of engagement with PV, “Responding” vs. “Viewing” in instructor-mediated classroom setting. We selected student samples for both our experiments such that they differed on the variable, “prior exposure” to active learning. Checks on sample selection were enforced through formal instructor interviews and observations of student behaviors in class as well as informal interviews with students. The claim of the student sample of experiment I having no “prior exposure” was confirmed through interviews with instructors and students and that for experiment II was confirmed through observational studies and instructor interview. Being a field experiment, we had to work with predetermined groups in both experiments. So, there was no chance of randomizing or matching the two groups in either experiment. However, we did prior testing on a criterion that was crucial to our study, i.e., programming skills. We found no statistically significant difference in a *t* test on programming skill marks. Student sample in the “Prediction” and “Viewing” groups in each experiment was drawn from the same sample population.

## Experiment II

Experiment II re-explored the three research questions with students who had prior training in active learning. In this section, we describe the sample characteristics and outline the process followed for in-class observation of student engagement distinct to experiment II along with the experimental results.

### Sample characteristics

To test the above hypotheses, a sample of 231 first-year undergraduate students was taken. These students had extensive prior exposure to active learning which was confirmed through a separate observational study of student behavior in the classroom that was carried out for a series of eight classes prior to this experiment (Kothiyal et al. 2013). In these classes, the instructor exposed the students to formal active learning for at least 20 min. It was found that in the first few classes, instructor had to explicitly spell out what students are expected to do in course of the active learning process. But after these few initial classes, we observed the students were cued in to their expected roles and did not require explicit instructions. This was also corroborated from interview with the instructor.

The first group of students was the “Prediction” group (*N* = 136; male = 120; female = 16), and the second group was the “Viewing” group (*N* = 95; male = 85; female = 10). The male:female ratio in the “Prediction” (7.5:1) and “Viewing” groups (8.5:1) was comparable. The groups were found to be equivalent on the basis of a prior quiz on programming skills using independent samples *t* test (*M*_experimental_ = 16.96 (SD = 5.86); *M*_control_ = 15.72 (SD = 6.09); *p* > 0.05).

### Procedure for in-class observation

The in-lecture observation of student behavioral engagement was carried out by a total of six observers. The inter-rater reliability with BOSS protocol was found to be good (Fleiss’s Kappa = 0.68). Each observer observed a random set of 20 students, twice during the prediction activity, for the “Prediction” group and the corresponding code segment for the “Viewing” group using the BOSS codes for active behavioral engagement. Individual students were observed for a fixed time interval of 5 s at a stretch. The total number of students thus observed per group was (20 × 6) = 120, and the total number of observations was 240 per group. The lecture was followed by the post-test and student affect and perception survey.

### Results

#### Behavioral engagement

The 240 observation codes in each group were categorized into engagement (active and passive) and non-engagement as per BOSS protocol codes. Both groups showed high behavioral engagement in the classroom with total engagement of the “Prediction” group (89.13 %) being higher than the “Viewing” group (80.41 %). We analyzed the engagement data further based on BOSS terminology and focused on the active engagement of students in the classroom. We found the “Prediction” group (23.33 %) to be more actively engaged than the “Viewing” group (9.58 %). Pearson’s chi-square (*χ* ^2^) test yielded a significant difference between the groups on active engagement (Table 5).Thus, the hypothesis, H _1___1_, is accepted. The prediction activity with PV does lead to significantly more active behavioral engagement in classroom than viewing alone.

**Table 5:** In-lecture active behavioral engagement

	Prediction group	Viewing group
Total observations	240	240
Active engagement observation frequency (percentage)	56 (23.33 %)	23 (9.58 %)
Chi-square results	*χ* ^2^ (1) = 4.42, *p* = 0.00	

#### Affect and perception of learning

The responses of both groups to the two-question survey, the same that was administered in experiment I, were analyzed. In Table 6, we report the total number of responses obtained per question in the survey. The analysis of the responses showed both groups highly recommended the respective instructional strategies used with the PV in their lecture though the “Prediction” group favored their strategy more than the “Viewing” group. For the “Prediction” group, 91.9 % recommended prediction with PV for use in the rest of the course and agreed that this strategy helped them learn. For the “Viewing” group, 87.4 % favored the use of visualization with instructor’s parallel commentary whereas 84.2 % agreed that this strategy helped them learn. We did Mann-Whitney *U* test with these survey responses on a five-point Likert scale and did not find a statistically significant difference in responses of the two groups on either question (Table 6). Thus, the response of the “Prediction” group students was higher but not significantly higher than the “Viewing” group. Hence, the hypothesis, H _1___2_, could not be accepted.

**Table 6:** Mann-Whitney results for affect and perception survey

Question	Group	Mean rank	*U*	*p* values
Q1. Instructional strategy used with visualization helped me learn	Prediction (*N* = 136)	113.24	6085	0.81
	Viewing (*N* = 91)	115.13		
Q2. I would recommend using the strategy for rest of the course	Prediction (*N* = 136)	117.21	5887	0.38
	Viewing (*N* = 92)	110.49		

#### Cognitive achievement

The experimental group was able to complete the post-test in half the time (10 min) allotted for the post-test (20 min). We found a statistically significant difference in the rate of problem solving (*p* = 0.00) in favor of the “Prediction” group (Table 7) with an effect size of 1.46. Thus, the hypothesis, (H_1___3a_) stating students have prior exposure to active learning will lead to higher rate of problem solving for the “Prediction” group than the “Viewing” group, is accepted. However, the average post-test scores did not exhibit a significant difference between the two groups (Table 7) with both groups exhibiting a good representation of high scores.

**Table 7:** Mann-Whitney *U* results for cognitive achievement

Dimension	Group	Standard deviation	Mean	*U*	*p* values
Rate of problem solving	Prediction (*N* = 136)	0.26	0.62	966.5	0.00
	Viewing (*N* = 95)	0.13	0.32		
Average post-test score	Prediction	2.55	6.18	6435	0.96
	Viewing	2.52	6.35		

## Discussion

### Combined analysis of experiment I and experiment II

Experiment I measures learning outcome from PV along the metrics of behavioral engagement, affect and perception, and cognitive achievement along the two engagement levels of “Responding” and “Viewing.” The students in this experiment did not have prior training in active learning. In experiment II, learning outcome was measured along the same metrics along the same engagement levels but the students had prior training in active learning. From the findings of both the experiments, we are able to conclude that active learning strategy of prediction with PV shows significantly active behavioral engagement in the “Prediction” group compared to the “Viewing” group for a medium complex topic in introductory programming course. This result is independent of students’ “prior exposure” to active learning in an instructor-mediated classroom.

Affect and perception of learning, measured through student responses on learning effectiveness and satisfaction, is higher for the “Prediction” group than the “Viewing” group. It was significantly so for students with no prior training in active learning but non-significant for students with prior training. This was probably because trained students in the “Viewing” group could not isolate out the difference in learning effectiveness and satisfaction of a passive strategy like simply watching the PV in a one-off class resulting in no significant difference in student perception between the “Prediction” and “Viewing” groups. We explored the affect and perception of learning further by comparing the responses of the “Prediction” groups across the two experiments when the groups were matched on the basis of common post-test marks. We found significantly higher positive response for the active learning strategy with PV for the student group that was exposed to active learning for the first time (Table 8). This result indicates the strong acceptance of the use of active learning strategies with PV among students in instructor-mediated college classrooms.

**Table 8:** Mann-Whitney *U* results for matched “Prediction” groups

Question	Group	Mean rank	*U*	*p* values
Q1. Instructional strategy used with visualization helped me learn	Experiment I (*N* = 39)	91.13	853	0.00
	Experiment II (*N* = 93)	56.17		
Q2. I would recommend using the strategy for rest of the course	Experiment I (*N* = 39)	91.27	847.5	0.00
	Experiment II (*N* = 93)	56.11		

**Fig. 6:**
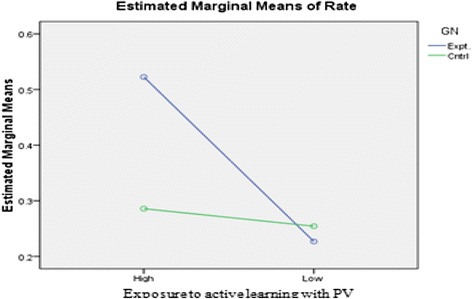
Cross-effect of 2 IVs—engagement level and prior exposure

Prior training in active learning appears to have an effect on cognitive achievement in terms of significantly increased rate of problem solving for students with prior training in active learning but no increase for students without prior training. To explore whether this variable “prior exposure” to active learning is indeed acting as a moderator variable on learning outcome besides engagement level with PV, we re-analyzed the data from both experiments together, by doing a factorial design across the two experiments with groups matched on basis of the common post-test marks. The analysis of the ANOVA results (Table 9) showed a significant interaction effect (*p* = 0.00) between the two independent variables (IVs)—engagement level with PV and prior exposure to active learning (Fig. 6). This result is informative for instructors planning to use PV in an instructor-mediated classroom. The rate of problem solving, indicating higher learning outcome, will be higher by using prediction with PV than simply playing the PV with parallel commentary, once the students get accustomed to the strategy. We could not do any detailed analysis for gender differences on learning outcome with PV since the number of female students in our sample was too small.

**Table 9:** Two-way ANOVA results of factorial design for the rate of problem solving

Dependent variable: rate of problem solving			
Independent variable	df	*F*	Significance
Engagement level	1	23.93	0.00
Prior exposure	1	58.45	0.00
Engagement level * prior exposure	1	38.08	0.00

### Discussion of our results

Our results show that by aligning the Prediction activity with our instructional objectives with the PV (based on theory-recommended strategy design components), we were able to achieve significant increase in behavioral engagement. We were also able to obtain increased cognitive achievement in terms of higher rate of problem solving through the strategy of “Prediction with PV” when students had prior training in active learning. We discuss our results in light of each of our research questions below.

- RQ1: Does prediction activity with PV (Responding) lead to higher levels of active behavioral engagement in an instructor-mediated classroom setting than simply viewing the visualization (Viewing) for a programming topic of medium complexity?

To answer this research question, we measured active behavioral engagement through the BOSS observation protocol. The results of experiments I and II revealed that irrespective of prior exposure to active learning, students in the “Prediction” group are significantly more actively engaged than those of the “Viewing” group. It may be argued that prediction with PV being an active instructional strategy, the result is obvious. But in light of study by Laakso et al. (2009) where students did not perform at the engagement level expected of them, this result is informative for instructors. The result shows that an active learning strategy can be implemented in lecture class with PV to ensure students are actively engaged, even if they do not have prior experience in active learning.

- RQ2: What are the student perceptions about learning effectiveness and satisfaction for the respective strategies used with PV in the classroom?

To answer the above research question, we administered a short survey to record student affect and perception about the active instructional strategy used with PV in their class. The results from both experiments indicate that students experiencing active learning for the first time in a lecture class perceive prediction with PV to be statistically significantly more learning effective and likeable than the “Viewing” group who simply watched the PV with parallel instructor commentary. Even for students with prior exposure to active learning, the “Prediction” group had better perception of learning effectiveness and satisfaction of the strategy compared to the “Viewing” group but not statistically significant. The probable reason for the non-significant difference is that the “Viewing” group, being conditioned to active learning, could not demarcate the effect of the passive strategy used with them in a one-off lecture. The take-away from this result for instructors is that active learning in the classroom is perceived to be learning effective and likeable by the students themselves, irrespective of prior exposure to active learning.

- RQ3: Does prediction activity with PV (Responding) lead to higher levels of cognitive achievement in an instructor-mediated classroom setting than simply viewing the visualization (Viewing) for a programming topic of medium complexity?

Cognitive achievement in the current study is measured in terms of the rate of problem solving and average post-test scores. From the results of both experiments, we found a significant difference in the rate of problem solving in favor of the “Prediction” group when students had prior exposure to active learning. But we got no significant difference in average post-test scores between the two groups in both the experiments. The possible reason could be the questions in the post-test were not able to capture the difference in learning.

Further analysis of our results led us to contemplate the effect of learning behavior of Indian students on the results. Empirical studies have shown positive correlation between culture and learning approaches (Sulkowski and Deakin 2009). Asian learners, including Indians, have been shown to possess learning characteristics such as relying on authority figures like instructors to impart knowledge, dislike of ambiguity and uncertainty, being less autonomous, and more obedient and conforming to rules as compared to Western students (Subramaniam, 2009). In our study, the students in both experiments were accustomed to instructor-driven “chalk & talk” classroom teaching. Their exposure to technological tools used within the classroom was restricted to powerpoint presentations. Nevertheless, these students are tech-savvy, being active users of social networking sites and mobile applications, and conversant with English. Thus, they were familiar with technological tools like visualization. The above characteristics of Indian students may explain why “prior exposure” to active learning emerged as an important moderating variable. The positive student perception of learning from “Prediction with PV” signals acceptance of the active learning strategy by the students. However, our results show that to derive cognitive achievement from the strategy, the students need to get accustomed to the execution process of the strategy and their role in it.

### Limitations of our study

One of the limitations of the current study is we did a post-facto factorial analysis of the results to study the moderating effect of “prior exposure” to active learning. The ideal condition would have been to set up a factorial design with “prior exposure” to active learning and engagement level as two independent variables and learning outcome from PV as the dependent variable. Also, this study was done on a single programming topic of medium complexity. To improve the generalizability of the results, we need to extend the experiments to other programming topics of medium complexity. To test if our results extend to topics of high complexity under the same condition set of instructor-mediated classrooms, further experiments need to be conducted. The third limitation is the way the cognitive achievement was measured. We were also not able to get any significant difference in average post-test scores. The post-test questions that tested cognitive achievement may not have been challenging enough to capture the difference in conceptual and procedural understanding between the “Prediction” and “Viewing” groups. This indicates more work is needed to identify the specific type of learning that occurs due to the visual aspect of PV and the corresponding questions that need to be included in the post-test to capture the learning from PV. The fourth limitation of the study is the length of the intervention which was 20 min for both groups. Increased intervention time with a second set of prediction activities with PV could have possibly led to greater knowledge acquisition for the “Prediction” group. Another limitation of the study was that being a field experiment, we had to work with predetermined groups. We could not get randomized or matched groups in either experiment. However, the groups were tested for equivalence on the crucial criterion of programming skills.

### Implications of our study

Our study is relevant for instructors in instructor-mediated classrooms in multiple ways. Such classrooms are characterized by limited availability of technological resources and predominance of one-way instructor-centered teaching (Kundi and Nawaz 2010). Here student interaction with visualization has to be mediated through the instructor. This study provides evidence of feasibility of implementation of active learning strategy with visualization in the aforesaid context. The study also shows the benefits that can be accrued from active learning strategy like prediction with PV in instructor-mediated classrooms even if the class size is large. Our results are a pointer to instructors that if complete cross-over to active learning with visualization appears challenging, they can start with subtle changes like integrating a small prediction activity with the visualization within their lecture. This can make a difference in learning outcome from visualization in instructor-mediated classrooms as compared to the traditional method of lecturing with visualization.

Additionally, the study makes the instructors aware of the condition set under which the current results will hold. For instance, our study shows that in a field setting of instructor-mediated college classrooms, active learning with visualization leads to increased learning outcome in terms of behavioral engagement and affect and perception of learning when compared to the lecture method, if the topic is of medium complexity. It also leads to increased cognitive achievement provided the learners have prior training in active learning, i.e., accustomed to the mechanics of the strategy. The study also addresses the reported problem on how to effectively integrate visualization in classrooms (Shaffer et al. 2011). It provides instructors with a pedagogically sound stepwise integration plan using the active learning strategy of “Prediction with PV” (Fig. 4).

Our study is also relevant to researchers in the field of teaching with visualization. The study identifies “prior exposure” to active learning as another moderating variable to consider while teaching with visualization. Existing studies have found increased cognitive achievement with increasing familiarity with the PV tool (Ben-Bassat Levy et al. 2003; Isohanni and Knobelsdorf 2011). We add to this literature by identifying “prior exposure” to active learning, i.e., increased familiarity with the active learning strategy leads to increased cognitive achievement with PV. Another contribution of the study is reporting increased learning outcome from visualization, along the metric of behavioral engagement, affect and perception of learning, and cognitive achievement, at the “Responding” level compared to the “Viewing” level with visualization where context is instructor-mediated classroom and topic complexity is medium. This result is interesting when contrasted with the results of existing studies with visualization along the axis of topic complexity. When teaching with visualization, topic complexity is an important moderating variable to consider. In the absence of clear definition of topic complexity, we relate topic complexity to the extent of prior knowledge required to comprehend the topic. Examples of programming topics of low, medium, and high complexity were given by Urquiza-Fuentes and Velázquez-Iturbide (2013) as infix operators, user-defined data types, and recursive data types, respectively. Prior studies give evidence that PV is not required for simple topics (Urquiza-Fuentes and Velázquez-Iturbide 2013; Ben-Bassat Levy et al. 2003). For highly complex topics, Urquiza-Fuentes and Velázquez-Iturbide (2013) found passive strategy of watching the visualization leads to better learning outcome than an active learning strategy of students constructing their own PV. Contradicting this result is the study by Jarc et al. (2000) which found students in the “Responding” level did better than those at the “Viewing” level for complex topics, though not significant For medium complexity programming topics, Urquiza-Fuentes and Velázquez-Iturbide (2013) found active learning strategy with PV was as beneficial as simply viewing. In our study, we have found active learning strategy with PV is significantly better than simply viewing the PV for medium complexity topic for cognitive achievement provided students had prior training in active learning. The major difference between these studies and ours is in the prevailing condition set. Comparing the study of Urquiza-Fuentes and Velázquez-Iturbide (2013) with our study, we found in the former study, students were given the intervention in self-study context with no instructor role. It involved problem solving with visualization in laboratory setting post a lecture class on the topic with students having direct access to the visualization. In our study, the intervention was in a lecture class setting where the instructor introduced them to the topic using the visualization and students did not have direct access to the visualization. Thus, through our study, we were able to determine the condition set under which “Responding” gives better results than “Viewing” for medium complexity programming topics in an instructor-mediated setting.

Another factor to consider for effective teaching with visualization is the alignment between instructional strategy used and the instructional objective with visualization (Boyle 2010). This alignment between strategy and objective can be achieved by incorporating a set of theory-recommended design components within the chosen strategy. Figure 3 shows how the chosen strategy of “Prediction with PV” was designed to incorporate the recommended design components mapped to our instructional objective with visualization which were (i) behavioral engagement in class and (ii) application of conceptual and procedural knowledge in problem solving. Thus, “Prediction with PV” incorporates design components like activating students’ prior knowledge and connecting it to new knowledge, raising cognitive conflict, providing procedural assistance among others to align with the stated objectives (Fig. 3). Our results show by incorporating these design components in our strategy, we were able to achieve significant increase in behavioral engagement. We were also able to obtain increased cognitive achievement in terms of higher rate of problem solving through the strategy of “Prediction with PV” when students had prior training in active learning. Thus, an active learning strategy is a means to operationalize the recommended design components.

Further analysis of our results led us to contemplate the effect of learning behavior of Indian students on the results. Empirical studies have shown positive correlation between culture and learning approaches (Sulkowski and Deakin 2009). Asian students, including Indians, are accustomed to follow instructor-driven teaching in the classroom. Indian students are tech-savvy, conversant with English, and familiar with technological tools like visualization. The above characteristics of Indian students may explain why “prior exposure” to active learning emerged as an important moderating variable. The positive student perception of learning from “Prediction with PV” signals acceptance of the active learning strategy by the students. However, our results show that to derive cognitive achievement from the strategy, the students need to build familiarity with the execution process of the strategy and their role in it.

## Summary and conclusion

The goal of this study was to contrast learning with PV in instructor-mediated classrooms between two different engagement levels with visualization—“Viewing” and “Responding.” From our field experiments, we found significant increase in active student behavioral engagement and their perception of learning in favor of the “Prediction” group for a programming topic of medium complexity. We also found significant increase in cognitive achievement for the “Prediction” group in terms of the rate of problem solving, provided students had prior training in active learning. Our study is relevant for both the researchers and the instructors. It identifies the learner characteristic of “prior exposure” to active learning as another moderating variable for researchers to consider. This study also provides evidence for instructors on feasibility of implementation of active learning strategy with visualization in instructor-mediated classrooms. The study also shows the benefits that can be accrued from active learning strategy like prediction with PV in the aforesaid context. Thus, our study reinforces the need for instructors to choose an active learning strategy with program and algorithm visualizations dependent on the set of conditions linked to their context like topic complexity and learner characteristic (prior exposure to active learning with visualization, achievement level). The current results are however applicable for programming topic of medium complexity. To make the results generalizable, further experiments are planned as future work to cover other medium complexity programming topics as also include topics of high complexity.
